# Insufficient uptake of systematic search methods in oncological clinical practice guideline: a systematic review

**DOI:** 10.1186/s12874-019-0818-5

**Published:** 2019-08-20

**Authors:** Chiara Trevisiol, Michela Cinquini, Aline S. C. Fabricio, Massimo Gion, Anne W. S. Rutjes

**Affiliations:** 1 Veneto Institute of Oncology IOV – IRCCS, Padua, Italy; 2 Unit of Systematic Reviews Methodology and Guidelines Production, Department of Oncology, Mario Negri Institute for Pharmacological Research IRCCS, Milan, Italy; 3 Regional Center for Biomarkers, Department of Clinical Pathology, Azienda ULSS 3 Serenissima, Campo SS Giovanni e Paolo 6777, 30122 Venice, Italy; 4 Institute of Social and Preventive Medicine (ISPM), University of Bern, Bern, Switzerland; 5 Institute of Primary Health Care (BIHAM), University of Bern, Hochschulstrasse 4, 3012 Bern, Switzerland

**Keywords:** Guideline, Systematic review, Reporting quality, Oncology, Reproducibility of result

## Abstract

**Background:**

The use of systematic review methods are widely recognized to be essential in the development of recommendations in clinical practice guidelines to prove their trustworthiness. The objective of this study was to assess the use of systematic search methods by authors of guidelines published in the oncology field.

**Methods:**

We analyzed 590 guidance documents identified in PubMed, NGC, GIN and web sites for guidelines in 2009–2015 in oncology. The main outcome measure used was incidence of guidance documents supported by a systematic search of the literature. In addition to descriptive analyses, logistic regression was used to evaluate if adequate search methods were explained by guideline characteristics.

**Results:**

Of 590 guidance documents included in the study, 305 (51.7%) declared the use of systematic search methods but only 168 (28.5%) applied methods meeting minimum standards for quality and provided sufficient details to allow classification. 164 (27.8%) guidance documents did not report any use of literature evaluation.

Guidance documents produced by a Government Agency in North America (OR 2.16, 95% CI 1.16–4.17) and those with a focused scope (OR 2.35, 95% CI 0.97–5.56) were positively associated with the use of systematic search methods. We found no association between the year of publication and use of systematic search methods.

**Conclusions:**

A relatively small number of guidance documents was informed by scientific evidence identified through adequate systematic search methods. We observed substantial room for improvement of applied methods and reporting, especially in documents with a broad focus, or those produced by professional societies or independent expert panels in other continents than North America.

**Electronic supplementary material:**

The online version of this article (10.1186/s12874-019-0818-5) contains supplementary material, which is available to authorized users.

## Background

Clinical practice guidelines (CPGs) are defined by Institute of Medicine (IOM) as statements that include recommendation intended to optimize patient care. They are informed by a systematic review of evidence and assessment of the benefit and harms of alternative care options [1]. This definition emphasizes the fact that clinical recommendations need to be based on the best available evidence evaluated through a systematic review of the medical literature.

According to the Cochrane collaboration handbook “A systematic review attempts to collate all empirical evidence that fits pre-specified eligibility criteria in order to answer a specific research question. It uses explicit, systematic methods that are selected with a view to minimizing bias, thus providing more reliable findings from which conclusions can be drawn and decisions made.” [2–4].

Similarly, the IOM and the Guidelines International Network (GIN) recommend that guideline developers use systematic review methods to identify and evaluate evidence related to the guideline topic [1, 5]. Selective assessment and unstructured appraisal of the literature may lead to recommendations that promote suboptimal or even harmful care [6]. Therefore, clinicians should consider whether recommendations in a guideline are relevant to their practice only after having verified that the guideline has been prepared according to proper methodological requirements.

A well-defined, transparent and reproducible search strategy along with grey literature searches and selection criteria to include evidence should be the starting point to achieve evidence-based CPGs [7].

The quality of practice guidelines has already been a source of concern in the past. In a previous overview Grilli and colleagues found that 87% of 431 analyzed guidelines did not report any information on the systematic search, although the proportion of guidelines reporting some form of search increased over time (2% in 1988–91 to 18% in 1996–98) [8].

The landscape for CPGs has significantly changed in the last two decades since the National Guideline Clearinghouse [9] was launched, the GIN International Guidelines Network [10] was established, and the Appraisal of Guidelines for Research and Evaluation (AGREE) consortium [11] and the Grading of Recommendations Assessment, Development and Evaluation (GRADE) working group [12] started their activities. GRADE methodology has impacted on CPGs definition and grading the quality of evidence, while quality and reporting for CPGs are widely evaluated by AGREE II tool. Nonewithstanding, we assisted to a world-wide proliferation of CPGs produced with different methods, and published in different reporting modalities by different panels.

Considering that systematic review of published evidence is an essential component in the methodological process of CPGs production, understanding how search strategies are used by Guideline Development Panels (GDPs) is important. Recently, three articles on reporting CPGs were published, but none of them assessed whether GDPs provided sufficient details to conclude that systematic searches were indeed performed [7, 13, 14].

In fact, from a methodological point of view a guideline is classified as Clinical Practice Guideline when it provides sufficient details to demonstrate that a systematic search and other systematic review steps were performed. In absence of any description of the search, or if the described strategy did not meet a set of minimum criteria, the document would be best referred to as “guidance document” [15].

The objective of this study, that is collateral to a national project aiming at synthesizing recommendations on traditional circulating tumor markers in solid tumors [16–18], was to assess the use of systematic search methods in CPGs in oncological field as a test context framework, and to determine which context variables were associated with adequate use.

## Methods

### Search strategy

A systematic literature search for existing guidelines in oncology published in English or Italian was undertaken using PubMed, National Guideline Clearinghouse, GIN Library databases and websites of 11 organizations and 61 Italian scientific societies producing CPGs. The search strategy was built with keywords related to cancer and guidelines. Full details are described in Additional file 1. Selection was independently performed by 3 examiners on the basis of the titles and abstracts of the identified records. A record was default included when at least 2 out of 3 examiners opted for inclusion. When only one examiner opted for inclusion, disagreement was resolved by discussion.

Full-text reports were retrieved for the potentially eligible documents. Every reasonable effort was made to locate related reports to included guidance documents online, that could provide additional information regarding the methodology used to generate the CPG recommendations.

### Selection criteria

Any document containing information and recommendations pertaining to diagnosis, work-up, management, treatment or follow-up of tumors on adult population was included. Moreover, documents had to meet all of the following eligibility criteria to be included:

- contain recommendations intended to optimize patient care and assist physicians, other practitioners and patients, to make decisions about appropriate health care for specific clinical circumstances;
- be produced under the auspices of, or endorsed by, government agencies or health care organizations, medical specialty associations or relevant professional societies;
- be produced, reviewed, reassessed for validity or updated between 2009 and 2015;
- being accessible in the public domain.

The following documents were excluded from the analysis:

- guidelines concerning malignancies in which traditional circulating tumor markers are not considered (e.g. brain tumors, soft tissue tumors, musculoskeletal tumors, non-melanoma skin cancers, hematological malignancies, hereditary/familial cancers);
- guidelines concerning malignancies in which traditional circulating tumor markers have no role in risk assessment or in the management (documents focusing only on screening, prevention, palliative care; documents focused on specific subgroups of patients, such as pregnant women, children, adolescents, homeless people);
- health technology assessment reports, systematic review and network meta-analyses;
- narrative reviews and comments or editorials accompanying the guidelines;
- guidelines developed and issued by an individual(s) not officially sponsored or supported by one of the organization types cited above.

Some reports were identified as *multiple reports* belonging to an unique guidance document if they were either ordered by the same organization/producer or produced during the same consensus meeting or if they were explicitly cited in the main guidance document. In these cases all the identified multiple reports were included to allow for a full appreciation of the guideline but they count as one document in our logistic analyses.

When the same guideline developer or GDP published guidance documents in different formats (summarized, abridged, or full version), or published in different journals or websites, we included the most recent unabridged version as main report.

### Data extraction

For each included guidance document, we collected the following characteristics.

- year of publication;
- scope of guidelines: broad (Multi-aspect, e.g. from diagnosis to metastatic disease management) or focused (centered on a specific clinical question or topic);
- type of organization/producer classified as Professional/Specialist Society, Government Agency, Independent Expert Panel, Other (Not-for-profit Organization, For-profit Organization, International Agency-i.e. WHO, Academic Institution);
- continent (considering the “seven-continent model”) classified as North America (United States, Canada), South America, Europe, Asia, Oceania, Africa, International (GDP members or producers from two or more continents);
- cancer type.

**Fig. 1:**
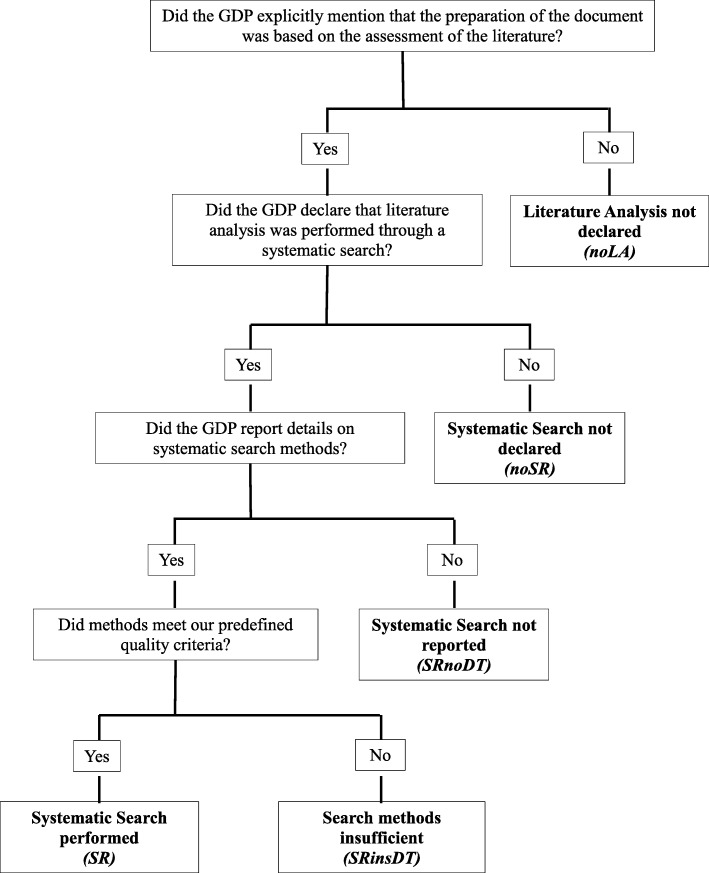
Guidance documents classification algorithm

Detailed information on degree of traceability of published evidence in the produced recommendations was collected following this pattern (Fig. 1):

- *Was the production of recommendations based on any literature analysis?*
- *If yes, did the GDP declare a systematic search of literature?* A very inclusive approach was used, for example, it was considered sufficient that the general review process was described as “comprehensive” or “extensive”, or that a database search was done.
- *If yes, did the GDP report details on the systematic review methods (*i.e. *biomedical database and/or web site search, keywords)?*

A systematic search strategy was considered sufficient and appropriate when all of the following three criteria were met:

- explicit reporting of the use of at least one biomedical database (e.g. MEDLINE);
- use of at least one additional source to retrieve citations, such as another biomedical database, screening of reference lists of included reports, personal communication with investigators or organizations;
- explicit description of the search terms used in bibliographic search(es).

When above criteria were met, the guidance document was classified as being informed by a systematic search method (SR). These criteria were inspired by the descriptions of Oxman and Guyatt in 1988 [19, 20]. A very similar approach was used by NICE in 2004 [21].

Two reviewers (CT, MC) have independently evaluated the guidance documents classifying them as documents:

- without explicit description of the use of literature analysis (noLA);
- with literature analysis but without reference to the use of systematic search methods (noSR);
- described to be based on one or more systematic reviews, not providing details to demonstrate it (SRnoDT);
- described to be based on one or more systematic reviews, with a search strategy not meeting our quality criteria (SRinsDT);
- described to be based on one or more systematic reviews, with a search strategy meeting our quality criteria (SR).

Any disagreement on the classification process was resolved by discussion or involvement of a third examiner (AR) until consensus was reached.

### Data analysis

Categorical variables were described as frequencies and percentages, considering the unit of analysis being the individual guidance document. Descriptive analyses were performed on all guidelines categories.

The association between several guideline characteristics and the type of literature evaluation was examined by univariate regression model analysis in two models. *Model 1* included the whole sample of guidelines, in order to compare documents declaring the use of systematic search methods (SR; SRinsDT; SRnoDT) with documents without declaration of systematic review or a literature analysis (noSR and noLA). *Model 2* was restricted to documents declaring the use of systematic search methods. In this model, we evaluated the association between guideline characteristics and the use of adequate search methods. The dependent variable in these logistic regression analyses was dichotomized in sufficient and appropriate (SR, as defined above) vs. less rigorous systematic search strategy (SRinsDT & SRnoDT). We presented the results of *Model 2* into two separate analyses, (SR vs SRinsDT) and (SR vs SRnoDT), respectively.

Documents produced from and published by the same organization, were included as a clustering variable in the regression analyses to obtain robust variances.

The independent variables evaluated in both models concerned:

- year of publication as continuous variable;
- type of organization/producer as categorical variable, defining *Government Agency* as the contrast;
- scope as categorical variable, defining *Broad* as the contrast;
- continent as categorical variable, defining *International* as the contrast.

Using a cut-off *p*-value of 0.05, we included candidate variables in a multivariable model to assess the predictors of the use of well conducted/reported systematic search methods.

Results are reported as odds ratios (ORs) with 95% CIs. Analyses were performed using SAS (Statistical Analysis System, SAS Institute Inc., Cary, NC, USA, Version 9.2) software. All tests were two-sided and *P* < 0.05 was used to determine statistical significance.

The study involved no human participants and required no ethical approval.

## Results

### Selection of guidelines

**Fig. 2:**
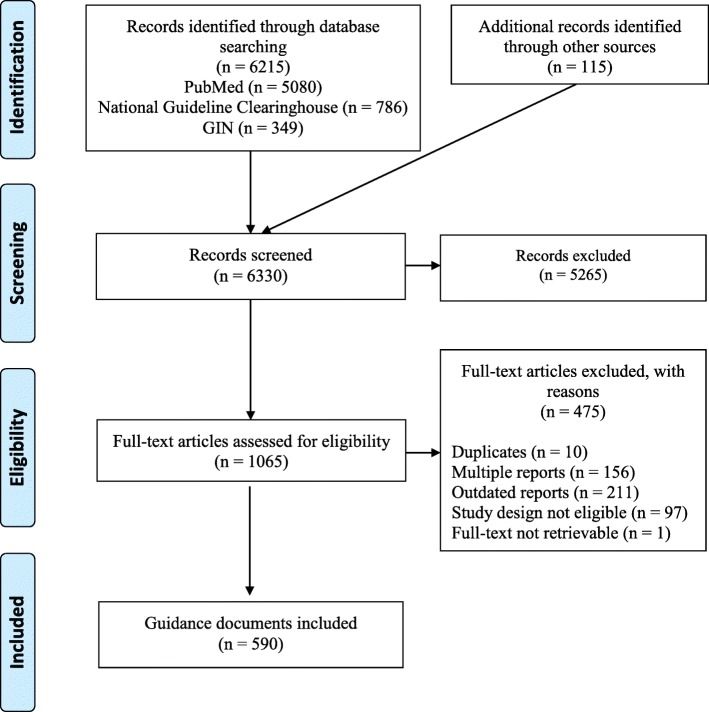
PRISMA 2009 Flow Diagram (modified)

Overall, of 6330 titles and abstract retrieved from the databases and websites searches, 1065 reports were identified as potentially eligible. Four hundred seventy-five were excluded after screening of the full-text. A final set of 590 guidance documents was identified. Full list is reported in Additional file 2. Figure 2 shows a modified PRISMA flow diagram [22].

### Guideline characteristics

Table 1 shows the characteristics of the included guidance documents. Overall, the number of guidelines increased during the years and their scope was focused in 346/590 (58.6%). Most of the documents were developed by Professional or Specialist Societies (378/590, 64.1%), followed by Government Agencies (108/590, 18.3%) and by Independent Expert Panels (80/590, 13.6%). They were published predominantly in North America (247/590, 41.9%) and Europe (222/590, 37.6%). No document published in Africa was identified. Guidelines were related to all tumor types, with the number of identified documents ranging from 2 documents for pleural neoplasm to 68 documents for prostatic cancer.

**Table 1:** Characteristics of 590 guidance documents by type of search methods

Characteristic	All (*n* = 590)	SR^b^ (*n* = 168)	SRinsDT^c^ (*n* = 111)	SRnoDT^d^ (*n* = 26)	noSR^e^ (*n* = 121)	noLA^f^ (*n* = 164)
Year of validity						
2009	41 (6.9%)	4 (3.6%)	2 (7.7%)	7 (5.8%)	16 (9.8%)	12 (7.1%)
2010	44 (7.5%)	6 (5.4%)	4 (15.4%)	9 (7.4%)	13 (7.9%)	12 (7.1%)
2011	69 (11.7%)	17 (15.3%)	3 (11.5%)	8 (6.6%)	26 (15.8%)	15 (8.9%)
2012	95 (16.1%)	22 (19.8%)	6 (23.1%)	22 (18.2%)	16 (9.8%)	29 (17.3%)
2013	115 (19.5%)	27 (24.3%)	4 (15.4%)	28 (23.1%)	22 (13.4%)	34 (20.3%)
2014	141 (23.9%)	20 (18.1%)	4 (15.4%)	30 (24.8%)	40 (24.4%)	47 (28.0%)
2015^a^	85 (14.4%)	15 (13.5%)	3 (11.5%)	17 (14.1%)	31 (18.9%)	19 (11.3%)
Scope						
Broad	244 (41.4%)	29 (26.1%)	15 (57.7%)	74 (61.2%)	77 (47.0%)	49 (29.2%)
Focused	346 (58.6%)	82 (73.9%)	11 (42.3%)	47 (38.8%)	87 (53.0%)	119 (70.8%)
Producer						
Government Agency	108 (18.3%)	10 (9.0%)	0	3 (2.5%)	6 (3.7%)	89 (53.0%)
Independent Expert Panel	80 (13.5%)	11 (9.9%)	5 (19.2%)	27 (22.3%)	25 (15.2%)	12 (7.0%)
Professional / Specialist Society	378 (64.1%)	83 (74.8%)	16 (61.6%)	88 (72.7%)	125 (76.2%)	66 (39.3%)
Other	24 (4.1%)	7 (6.3%)	5 (19.2%)	3 (2.5%)	8 (4.9%)	1 (0.6%)
Continent						
Asia	52 (8.8%)	10 (9.0%)	6 (23.1%)	7 (5.8%)	24 (14.6%)	5 (2.9%)
Oceania	14 (2.4%)	3 (2.7%)	1 (3.8%)	3 (2.5%)	2 (1.2%)	5 (2.9%)
Europe	222 (37.6%)	18 (16.2%)	10 (38.5%)	78 (64.4%)	76 (46.3%)	40 (23.8%)
North America	247 (41.9%)	71 (64.0%)	3 (11.5%)	19 (15.7%)	45 (27.5%)	109 (64.9%)
South America	7 (1.2%)	2 (1.8%)	0	3 (2.5%)	1 (0.6%)	1 (0.6%)
International	48 (8.1%)	7 (6.3%)	6 (23.1%)	11 (9.1%)	16 (9.8%)	8 (4.9%)
Neoplasm						
Anal cancer	9 (1.5%)	3 (2.7%)	0	1 (0.8%)	2 (1.2%)	3 (1.8%)
Biliary cancer	12 (2.0%)	3 (2.7%)	6 (23.1%)	2 (1.7%)	1 (0.6%)	0
Bladder cancer	27 (4.6%)	9 (8.1%)	2 (7.8%)	4 (3.3%)	5 (3.1%)	7 (4.2%)
Breast cancer	45 (7.6%)	8 (7.2%)	3 (11.5%)	5 (4.1%)	14 (8.5%)	15 (8.9%)
Cervical cancer	18 (3.0%)	4 (3.6%)	2 (7.8%)	3 (2.5%)	4 (2.4%)	5 (3.0%)
Colorectal cancer	52 (8.8%)	12 (10.8%)	1 (3.8%)	12 (9.9%)	14 (8.5%)	13 (7.7%)
Endometrial cancer	17 (2.9%)	5 (4.5%)	0	3 (2.5%)	3 (1.8%)	6 (3.5%)
Esophageal cancer	15 (2.5%)	2 (1.8%)	0	4 (3.3%)	3 (1.8%)	6 (3.5%)
Gastric cancer	18 (3.1%)	2 (1.8%)	0	4 (3.3%)	9 (5.5%)	3 (1.8%)
Germ cell tumor	3 (0.5%)	0	0	1 (0.8%)	1 (0.6%)	1 (0.6%)
Head and neck cancer	16 (2.7%)	0	1 (3.8%)	5 (4.1%)	7 (4.3%)	3 (1.8%)
Hepatocellular carcinoma	29 (4.9%)	4 (3.6%)	2 (7.8%)	7 (5.8%)	10 (6.1%)	6 (3.5%)
Incidentaloma	5 (0.9%)	2 (1.8%)	0	0	2 (1.2%)	1 (0.6%)
Lung cancer	55 (9.3%)	10 (9.1%)	1 (3.8%)	7 (5.8%)	12 (7.3%)	25 (14.9%)
Melanoma	20 (3.4%)	0	1 (3.8%)	4 (3.3%)	8 (4.9%)	7 (4.2%)
Mesothelioma	8 (1.4%)	0	1 (3.8%)	1 (0.8%)	3 (1.8%)	3 (1.8%)
Metastatic ab initio	4 (0.7%)	4 (3.6%)	0	0	0	0
NETs	23 (3.9%)	3 (2.7%)	1 (3.8%)	9 (7.5%)	7 (4.3%)	3 (1.8%)
Ovarian cancer	28 (4.8%)	4 (3.6%)	1 (3.8%)	7 (5.8%)	7 (4.3%)	9 (5.4%)
Pancreatic cancer	17 (2.9%)	2 (1.8%)	1 (3.8%)	3 (2.5%)	6 (3.7%)	5 (3.0%)
Penile cancer	5 (0.9%)	2 (1.8%)	0	1 (0.8%)	1 (0.6%)	1 (0.6%)
Pleural disease	2 (0.3%)	0	0	0	0	2 (1.2%)
Prostatic cancer	68 (11.5%)	15 (13.5%)	2 (7.8%)	12 (9.9%)	18 (11.0%)	21 (12.5%)
Renal cancer	23 (3.9%)	5 (4.5%)	0	6 (5.0%)	6 (3.7%)	6 (3.5%)
Testicular cancer	14 (2.4%)	1 (0.9%)	1 (3.8%)	5 (4.1%)	3 (1.8%)	4 (2.4%)
Thyroid cancer	25 (4.2%)	7 (6.3%)	0	6 (5.0%)	5 (3.1%)	7 (4.2%)
Unknown primary site	4 (0.7%)	0	0	1 (0.8%)	2 (1.2%)	1 (0.6%)
Multiple cancer	25 (4.2%)	4 (3.6%)	0	8 (6.6%)	11 (6.7%)	2 (1.2%)
Other	3 (0.5%)	0	0	0	0	3 (1.8%)

^a^From January, 1st to July, 15th

^b^SR = systematic search methods meeting our quality criteria; ^c^SRinsDT = described systematic search methods did not meet our quality criteria; ^d^SRnoDT = use of systematic search described, but no details provided; ^e^noSR = literature analysis described, but not as systematic; ^f^noLA = no reference to any type of literature analysis

One hundred sixty four (27.8%) documents did not declare any literature analysis, while 72.2% (*n* = 426) based the production of recommendations on literature evaluation. Of these 121 (28.4%) did not report a systematic literature review.

In 305 guidelines the GDP declared to have performed a systematic search of the literature. In this subgroup, 168/305 (55.1%) met our quality standards, 111/305 (36.4%) did not (i.e. only one or two quality standards criteria out of three were met) and 26/305 (8.5%) did not provide any detail on the search methods used.

Among the 111 guidance documents performing a systematic search not meeting our quality standards, 12/111 (10.8%) reported the biomedical database search only, 36/111 (32.4%) reported details on biomedical database search and other sources, while 60/111 (54.1%) reported details on biomedical database search and keywords. Two guidance documents reported only the keywords and 1 reported keywords and other sources but not the biomedical database searched.

### Regression analyses

Results are reported in Table 2

**Table 2:** Results of the univariate logistic regression analyses

Independent variable	Reference category	Effect	Odds Ratio (95% CI) *Model 1* – *n* = 590 (SR, SRinsDT, SRnoDT vs noSR, noLA)^a^	Odds Ratio (95% CI) *Model 2* – *n* = 305 (SR vs SRinsDT)^a^	Odds Ratio (95% CI) *Model 2* – *n* = 305 (SR vs SRnoDT)^a^
Year of validity					
(continuous variable)	–	Year of validity	0.98 (0.91–1.06)	1.01 (0.90–1.13)	1.17 (0.95–1.45)
Year of validity	2009	2010	1.28 (0.67–2.43)	1.50 (0.47–4.76)	2.00 (0.40–10.00)
		2011	1.32 (0.57–3.03)	3.40 (0.99–11.73)	1.20 (0.32–4.58)
		2012	1.92 (0.12–3.23)	2.28 (0.85–6.09)	1.24 (0.23–6.75)
		2013	1.66 (1.02–2.70)	2.38 (0.79–7.22)	0.71 (0.16–3.17)
		2014	1.30 (0.72–2.32)	1.28 (0.47–3.48)	0.51 (0.21–1.24)
		2015	0.99 (0.52–1.89)	2.37 (0.67–8.36)	0.95 (0.11–8.36)
Scope	Broad	Focused	2.57 (1.47–4.55)	0.86 (0.56–1.32)	3.33 (1.20–9.12)
Producer	Government Agency	Independent expert panel	0.05 (0.02–0.10)	0.12 (0.04–0.38)	0.02 (0.00–0.11)
		Professional or specialist society	0.07 (0.03–0.15)	0.09 (0.05–0.16)	0.03 (0.00–0.15)
		Other	0.11 (0.04–0.39)	0.02 (0.01–0.14)	0.01 (0.00–0.02)
Continent	International	Asia	0.87 (0.43–1.75)	0.44 (0.18–1.05)	0.63 (0.16–2.46)
		Oceania	2.31 (0.71–7.14)	1.46 (0.37–7.45)	3.75 (0.34–40.84)
		Europe	0.57 (0.31–1.03)	1.94 (0.76–6.03)	3.00 (0.97–9.33)
		North America	3.68 (2.22–5.88)	1.34 (0.53–3.11)	27.25 (6.01–123.48)
		South America	0.96 (0.25–3.70)	0.33 (0.01–5.57)	0.15 (0.01 - ∞)

ORs larger than 1 refer to increased use of systematic search methods in model 1 and increased use of search methods meeting our quality criteria in model 2

^a^Abbreviations are explained in Table 1

#### Model 1

The univariate models including all 590 guidance documents. Despite the increased number of guidelines produced over the years, the ratio between documents declaring and those not declaring the use of systematic search methods remained unchanged (OR 0.98, 95% CI 0.91–1.06). No trend was detected over the year (p for trend = 0.732).

The probability of declaring a systematic search method was strongly influenced by the scope of the guidance document. Documents with a focused scope were more likely to report the use of systematic search methods (OR 2.57, 95% CI 1.47–4.55).

Taking government agencies as a reference, all documents developed by other guideline producers were less likely to declare the use a systematic search. Guidance documents produced by an independent expert panel have the highest probability of not declaring any systematic search (OR 0.05, 95% CI 0.02–0.10).

The only continent associated with a significant higher uptake of systematic search methods was North America with an OR of 3.68 (95% CI 2.22–5.88). This result was confirmed when considering USA and Canada as distinct nationalities (data not shown).

#### Model 2

The univariate models included guidance documents explicitly mentioning the use of systematic searches (*n* = 305). Guidelines having a focused scope and being produced in North America more often met our quality criteria for adequate search methods (OR 3.33; 95% CI 1.20–9.12 and OR 27.25; 95% CI 6.01–123.48 respectively). When we used documents meeting some but not all of our quality criteria as comparator, the evidence was less conclusive (OR 0.86; 95% CI 0.56–1.32 and OR 1.34; 95% CI 0.53–3.11 respectively). Producers other than government agency were less likely to meet all or part of our quality criteria for adequate search strategies.

Table 3 presents results from the multivariable regression analyses, confirming those from univariate models. A systematic search was more likely to be declared if documents were produced in North America, by a government agency and with a focused scope. *In model 2,* using documents not explaining search methods as comparator, the multivariate analysis showed that documents produced in North America, by a Government agency, with a focused scope were more likely to use sufficient methods. Using documents meeting some but not all of the quality criteria as comparator, we found no statistically significant associations, except for non-governmental bodies that remain associated with lower probabilities of using sufficient search methods.

**Table 3:** Results of the multivariable regression analyses

Independent variable	Reference category	Effect	Odds Ratio (95% CI) *Model 1* – *n* = 590 (SR, SRinsDT, SRnoDT vs noSR, noLA)^a^	Odds Ratio (95% CI) *Model 2* – *n* = 305 (SR vs SRinsDT)^a^	Odds Ratio (95% CI) *Model 2* – *n* = 305 (SR vs SRnoDT)^a^
Scope					
Year of validity	2009	2010	1.59 (0.58–4.35)		
		2011	2.04 (0.69–5.88)		
		2012	2.70 (1.23–5.88)		
		2013	1.92 (0.89–4.35)		
		2014	1.45 (0.54–3.85)		
		2015	1.67 (0.56–5.00)		
	Broad	Focused	2.35 (0.97–5.56)	1.04 (0.49–2.20)	3.48 (0.89–13.61)
Producer	Government Agency	Independent expert panel	0.05 (0.03–0.10)	0.10 (0.03–0.31)	0.13 (0.00–0.96)
		Professional or specialist society	0.09 (0.04–0.16)	0.08 (0.04–0.15)	0.07 (0.00–0.33)
		Other	0.13 (0.04–0.42)	0.01 (0.00–0.05)	0.01 (0.00–0.03)
Continent	International	Asia	0.90 (0.35–2.86)	0.40 (0.11–1.53)	0.85 (0.16–4.53)
		Oceania	1.00 (0.34–4.17)	2.24 (0.12–40.82)	6.11 (0.51-∞)
		Europe	0.50 (0.23–1.05)	1.15 (0.40–3.29)	2.89 (0.49–17.09)
		North America	2.16 (1.16–4.17)	0.50 (0.19–1.29)	17.43 (2.28–133.44)
		South America	1.30 (0.23–10)	0.37 (0.02–6.29)	0.44 (0.02-∞)

ORs larger than 1 refer to increased use of systematic search methods in model 1 and increased use of search methods meeting our quality criteria in model 2

^a^Abbreviations are explained in Table 1

## Discussion

In the present study we analyzed guidance documents published over a 5 year period in oncology to evaluate the use of systematic searches, the adequateness of these searches and the context variables that may be associated with adequate use.

We found that, although a majority of guidance documents is informed by literature analyses, only a minority met pre-defined criteria for systematic search methods. Still about a quarter of guidance documents did not perform a literature analysis at all to produce recommendations. Moreover, we found no evidence that the use of systematic search methods or its adequateness improved over time since 2009 to 2015. Guidance documents with a focused scope produced by governmental bodies, especially in North America were most likely to use sufficient search methods.

The formation of a guideline panel with strong clinical and methodological background is paramount for developing and using guidelines. Any guideline being considered for guiding clinical practice, endorsement or adaptation should be informed by appropriate and up-to-date systematic reviews of the literature. The adequateness of a search strategy is determined by several factors and in this study, we only stipulated three minimum criteria that a search strategy should meet: searching at least one bibliographic database, searching at least one additional source and reporting keywords. We found that the vast majority (7 out of 10) of guidance documents did not meet this limited set of quality criteria. Within the set declaring the use of systematic search methods, still 4 out of 10 did not meet our criteria so that we observed room for improvement in both the use and reporting of adequate search strategies.

Our regression analyses showed strong positive associations between declared use of systematic search methods and guideline production by government agencies and in North America. These results are possibly due to involvement and funding from public institutions and perhaps the strong uptake of evidence based methods in this area. On the other hand, the positive associations found between declared use of systematic search methods and guideline production in North America seems not due to the fact that both the USA and Canada are high income countries. In fact, guidance documents from two other continents (Europe and Oceania) are all produced in high income countries and 551 out of 590 examined guidance documents are produced by high income countries. Despite the availability of guidance tools for guidelines development and assessment (GRADE, AGREE, GIN and IOM standards) being progressively disseminated in the past decade, we did not detect an improved uptake of literature analyses or systematic search methods in the past 5 year. Notwithstanding the evidence, the scientific community progressively assumes systematic searches to be “obviously done” in comprehensive guidance documents that were produced over the past years. The more recent reporting tools for practice guidelines in health care can be taken as an example [7, 13]. The RIGHT Working Group developed a checklist that includes 22 items considered essential for proper reporting of CPGs; as concerns evidence, the items 11a and 11b query how systematic reviews were done, not if they were done. One could reason that a document omitting descriptions of a systematic review process is a guidance document rather than a CPG, but still our findings are alarming. Guidance documents not meeting basic search requirements are used to drive appropriate clinical decisions and second, in spite of intentions and advice of GDPs, guidance documents are more and more commonly used as formal documents in specific fields such as health policies and legal litigations, without adequate implementation processes in clinical practice.

Every GDP can choose a reporting format based on their specific audience [13]. Taking lung cancer as an example, the National Institute for Health and Care Excellence reported all clinical questions in a unique document [23]. Conversely, the Cancer Care Ontario [24] produced separate guidelines for every clinical condition (diagnosis, initial work-up, therapy monitoring, etc) whereas the American College of Chest Physicians published different reports in an unique journal supplementary number [25]. It was therefore challenging to classify multiple reports in order to fit with the clinical questions fixed in the present study for individual malignancies and for specific clinical conditions (diagnosis, initial work-up, therapy monitoring, etc). The adoption of different classifications of multiple reports leads to different counts of examined documents.

### Strengths and limitations of this study

A strength of the present study was the use of a duplicate and independent processes throughout the project as well as the use of systematic and transparent method in searching, screening and collecting data. Moreover, we reported on a representative sample of guidance documents in oncology, without restricting to the type of clinical questions addressed.

Our study also presents some limitations. Regarding applicability, although oncology is a crucial health sector, it remains to be investigated if our results can be transferred to other healthcare areas. In addition, as the majority of the identified guidelines were developed in Europe and North America, both high-income countries, our sample may not be representative to middle or low income countries. Our selection may be biased by the English / Italian language limit that was imposed in the selection and by the type of bibliographic databases searched. We however reason that it is unlikely that our findings would be more favorable if our search methods would have been broader. To clarify, Italian national guidelines were included in the present study because it was branded from a national project in Italy aiming at extensively evaluating and synthesizing recommendations on tumor markers [16–18]. However, the number of Italian guidelines identified pose a small percentage of both the European and the total guidelines evaluated (*n* = 24/222, 11%; *n* = 24/590, 4%, respectively). Poor reporting of search strategies may have led to misclassifications. This type of bias occurs in any methodological assessment of any type of research, but we attempted to avoid misclassifications as much as possible by actively searching for multiple reports to identified guidance documents that might contain additional information on search methods. Lastly, although a relatively large set of documents was identified, the number of documents declaring the use of systematic search methods was rather small. Consequently, the evaluation of adequateness of applied methods resulted in typically wide confidence intervals around the summary estimates so that the magnitude of associations are uncertain.

### In context with previous studies and policy implications

In the present study we confirm and expand similar findings from other studies in more restricted areas. Grilli et al. reported in 2000 that the majority of documents published as CPGs do not provide evidence of the use of systematic review methods [8]. In 2008, Somerfield showed that few professional organizations rely on systematic reviews as the basis for oncology guidelines [26]. Reames et al., who evaluated oncology guidelines for cancer type with high mortality, noted a poor compliance with IOM standard’s systematic review criteria [27].

Findings of the present study indicate two issues to be considered in future guideline preparation: the first issue concerns the policy of guideline development, which is best improved at an organizational level and at the level of steering groups which have roles and responsibilities in managing guideline production; the second is focusing on improving methodology and/or reporting, which is best addressed in the GDPs which include both clinical experts and methodologists.

## Conclusion

In this systematic review of guidance documents in the field of oncology, we found that there still is substantial room for improvement of the uptake of adequate systematic search methods in the development of guidelines. Only a relatively small proportion of guidance documents was informed by scientific evidence identified through adequate systematic search methods. Best strategies to obtain improvement will vary across settings and continents but may involve educational interventions to sensitize CPGs producers to improve quality of methods and reporting but also governmental interventions to promote adequate methods with a call to scientific organizations to reduce the number of documents that are based on expert opinions without systematically evaluating the scientific literature.

## Additional files

Additional file 1: Full details of the search strategies. Systematic search strategy for identification of clinical practice guidelines; list of professional health organizations and government agencies searched for guidance documents; list of scientific Italian health societies or associations searched for guidance documents. (DOCX 25 kb)

Additional file 2: Guidance documents list. List and classification of all the included Guidance Documents. (XLSX 83 kb)

## Data Availability

The datasets used and analyzed during the current study are available from the corresponding author.

## Abbreviations

CPGs: Clinical practice guidelines
GDPs: Guideline Development Panels
GIN: Guidelines International Network
IOM: Institute of Medicine
noLA: no reference to any type of literature analysis
noSR: literature analysis described, but not as systematic
ORs: odds ratios
SR: systematic search methods meeting our quality criteria
SRinsDT: described systematic search methods did not meet our quality criteria
SRnoDT: use of systematic search described, but no details provided
