# Supplementary material for: Insufficient uptake of systematic search methods in oncological clinical practice guideline: a systematic review
Source: BMC Med Res Methodol. 2019 Aug 20;19:180. doi: 10.1186/s12874-019-0818-5 (PMC6702747; doi:10.1186/s12874-019-0818-5)
Supplement: Supplementary file 1 — Full details of the search strategies. Systematic search strategy for identification of clinical practice guidelines; list of professional health organizations and government agencies searched for guidance documents; list of scientific Italian health societies or associations searched for guidance documents. (DOCX 25 kb) [file 12874_2019_818_MOESM1_ESM.docx]

**Additional File 1: Full details of the search strategies**

**Systematic search strategy for identification of clinical practice guidelines.**

| Query | Search terms |
| --- | --- |
| *Pubmed* (<https://www.ncbi.nlm.nih.gov/pubmed>http://www.ncbi.nlm.nih.gov/pubmed) | |
| 1 | guidance[ti] OR guideline*[ti] OR recommendation*[ti] OR consensus[ti] OR practice guideline[pt] OR guideline[pt] |
| 2 | consensus development conference[pt] OR consensus development conferences as topic[MeSH] OR consensus[MeSH] |
| 3 | Query 1 OR 2 |
| 4 | neoplasms[MeSH Terms] OR carcinoma[MeSH Terms] OR cancer[tiab] OR neoplasm[tiab] OR neoplasms[tiab] OR tumor[tiab] OR tumour[tiab] OR tumors[tiab] OR tumours[tiab] OR carcinoma[tiab]) |
| 5 | Query 3 AND 4 |
| 6 | Limit 5 to English OR Italian Language, and Publication date from 2009/01/01 to 2015/07/16 |
| *GIN Library* (<http://www.g-i-n.net/library>) | |
| 1 | neoplasms OR cancer OR carcinoma OR tumors OR cancers OR tumor OR tumours OR adenocarcinoma OR neoplasia OR malignancy OR malignancies OR carcinomas OR tumour  Search date: 2015/01/16 |
| *National Guideline Clearinghouse* (<http://www.guidelines.gov>) | |
| 1 | NGC Browse: by topic – Disease/Condition – Diseases – Neoplasms  Search date: 2015/07/10 |

**List of professional health organization and governmental agencies .**

| CCO | Cancer Care Ontario (Canada) <https://www.cancercare.on.ca> |
| --- | --- |
| ACP | Association of Cancer Physicians (U.S.A) <http://www.acponline.org/> |
| ASCO | American Society of Clinical Oncology (U.S.A) <http://www.asco.org> |
| NCCN | National Comprehensive Cancer Network® (U.S.A) <http://www.nccn.org> |
| NHMRC | National Health and Medical Research Council (Australia) <http://www.nhmrc.gov.au/guidelines/index.htm> |
| NICE | National Institute for Health and Clinical Excellence (U.K.) <http://guidance.nice.org.uk> |
| NZGG | New Zealand Guidelines Group (New Zealand) <http://www.nzgg.org.nz> |
| SIGN | Scottish Intercollegiate Guidelines Network (U.K.) <http://www.sign.ac.uk> |
| USPSF | U.S. Preventive Services Task Force (U.S.A) <http://www.uspreventiveservicestaskforce.org/> |
| SNLG | Sistema Nazionale Linee Guida (Italy) <http://www.snlg-iss.it/> |
| AGENAS | <http://www.agenas.it/le_linee_guida.html> |

**List of scientific Italian health societies or associations.**

| AIMF | [Accademia Italiana dei Medici di Famiglia](http://www.dimf.it/aimf/aimf.htm) |
| --- | --- |
| ACOI | [Associazione Chirurghi Ospedalieri Italiani](http://www.fism.it/soci/detail.jhtml;jsessionid=59D6723B7947306AE4313786AE5C269A?id=6) |
| ADOI | [Associazione Dermatologi Ospedalieri Italiani](http://www.fism.it/soci/detail.jhtml;jsessionid=59D6723B7947306AE4313786AE5C269A?id=8) |
| AGICO | [Associazione Ginecologi Consultoriali](http://www.telemacus.it/agico) |
| AGUI | Associazione Ginecologi Universitari Italiani |
| AIDA | [Associazione Italiana Dermatologi Ambulatoriali](http://www.aida.it/) |
| AIOM | [Associazione Italiana di Oncologia Medica](http://www.fism.it/soci/detail.jhtml;jsessionid=59D6723B7947306AE4313786AE5C269A?id=19) |
| AIOT | [Associazione Italiana di Oncologia Toracica](http://www.fism.it/soci/detail.jhtml;jsessionid=59D6723B7947306AE4313786AE5C269A?id=236) |
| AIPaC MeM | [Associazione Italiana di Patologia Clinica e di Medicina Molecolare](http://www.fism.it/soci/detail.jhtml;jsessionid=59D6723B7947306AE4313786AE5C269A?id=20) |
| AIRO | [Associazione Italiana di Radioterapia Oncologica](http://www.fism.it/soci/detail.jhtml;jsessionid=59D6723B7947306AE4313786AE5C269A?id=244) |
| AIGO | [Associazione Italiana Gastroenterologi & Endoscopisti Digestivi Osp](http://www.fism.it/soci/detail.jhtml;jsessionid=59D6723B7947306AE4313786AE5C269A?id=68) |
| AIMEF | [Associazione Italiana Medici di Famiglia](http://www.aimef.org/) |
| AISF | [Associazione Italiana Per lo Studio del Fegato](http://www.fism.it/soci/detail.jhtml;jsessionid=59D6723B7947306AE4313786AE5C269A?id=29) |
| AIPO | [Associazione Italiana Pneumologi Ospedalieri](http://www.fism.it/soci/detail.jhtml;jsessionid=59D6723B7947306AE4313786AE5C269A?id=31) |
| AME | [Associazione Medici Endocrinologi](http://www.fism.it/soci/detail.jhtml;jsessionid=59D6723B7947306AE4313786AE5C269A?id=229) |
| AOGOI | [Associazione Ostetrici Ginecologi Ospedalieri Italiani](http://www.fism.it/soci/detail.jhtml;jsessionid=59D6723B7947306AE4313786AE5C269A?id=247) |
| AOOI | [Associazione Otorinolaringologi Ospedalieri Italiani](http://www.aooi.it/) |
| AUORL | [Associazione Universitaria Otorinolaringolog](http://www.auorl.it/) |
| AURO | [Associazione Urologi ospedalieri Italiani](http://www.fism.it/soci/detail.jhtml;jsessionid=59D6723B7947306AE4313786AE5C269A?id=49) |
| CseRMEG | [Centro Studi e Ricerca in Medicina Generale](http://www.csermeg.it/) |
| FADOI | [Federazione delle Associazioni Dirigenti Ospedalieri Internisti](http://www.fism.it/soci/detail.jhtml;jsessionid=59D6723B7947306AE4313786AE5C269A?id=62) |
| FISM | [Federazione delle Società Medico - Scientifiche Italiane](http://www.sameint.it/fism/index.html) |
| FIMMG | [Federazione Italiana Medici di Famiglia](http://www.fimmg.org/) |
| FISMELAB | [Federazione Italiana Società Medicine di Laboratorio](http://www.fism.it/soci/detail.jhtml;jsessionid=59D6723B7947306AE4313786AE5C269A?id=71) |
| GIMBE | [Fondazione GIMBE](http://www.fism.it/soci/detail.jhtml;jsessionid=59D6723B7947306AE4313786AE5C269A?id=76) |
| GOIM | [Gruppo Oncologico dell'Italia Meridionale](http://www.fism.it/soci/detail.jhtml;jsessionid=59D6723B7947306AE4313786AE5C269A?id=79) |
| ISPLAD | [International Italian Society of Plastic Regenerative and Oncologic Dermatology](http://www.fism.it/soci/detail.jhtml;jsessionid=59D6723B7947306AE4313786AE5C269A?id=219) |
| NITp | [Nord Italia Transplant](http://www.fism.it/soci/detail.jhtml;jsessionid=59D6723B7947306AE4313786AE5C269A?id=85) |
| SIAPEC | [Società Italiana di Anatomia Patologica eCitodiagnostica](http://www.siapec.it/) |
| SIBIOC | [Società Italiana di Biochimica Clinica e Biologia Molecolare Clinica](http://www.fism.it/soci/detail.jhtml;jsessionid=59D6723B7947306AE4313786AE5C269A?id=107) |
| SIC | [Società Italiana di Cancerologia](http://sos.unige.it/sic/index.html) |
| SIC | [Società Italiana di Chirurgia](http://www.fism.it/soci/detail.jhtml;jsessionid=59D6723B7947306AE4313786AE5C269A?id=115) |
| SICCR | [Società Italiana di Chirurgia Colo-Rettale](http://www.fism.it/soci/detail.jhtml;jsessionid=59D6723B7947306AE4313786AE5C269A?id=243) |
| SICO | [Società Italiana di Chirurgia Oncologica](http://sos.unige.it/sico/sico.html) |
| SIC | [Società Italiana di Citologia](http://www.fism.it/soci/detail.jhtml;jsessionid=59D6723B7947306AE4313786AE5C269A?id=117) |
| SIDeMaST | [Società Italiana di Dermatologia Medica, Chirurgica, Estetica e delle Malattie Sessualmente Trasmesse](http://www.fism.it/soci/detail.jhtml;jsessionid=59D6723B7947306AE4313786AE5C269A?id=223) |
| SIEOG | [Società Italiana di Ecografia Ostetrica e Ginecologica e Metodologie Biofisiche](http://www.fism.it/soci/detail.jhtml;jsessionid=59D6723B7947306AE4313786AE5C269A?id=218) |
| SIE | [Società Italiana di Endocrinologia](http://www.dfc.unifi.it/sie) |
| SIED | [Società Italiana di Endoscopia Digestiva](http://www.fism.it/soci/detail.jhtml;jsessionid=59D6723B7947306AE4313786AE5C269A?id=130) |
| SIFO | [Società Italiana di Farmacia Ospedaliera e dei servizi farmaceutici delle Az. Sanitarie](http://www.fism.it/soci/detail.jhtml;jsessionid=59D6723B7947306AE4313786AE5C269A?id=239) |
| SIGE | [Società Italiana di Gastroenterologia](http://www.fism.it/soci/detail.jhtml;jsessionid=59D6723B7947306AE4313786AE5C269A?id=134) |
| SIGOT | [Società Italiana di Geriatria Ospedale e Territorio](http://www.fism.it/soci/detail.jhtml;jsessionid=59D6723B7947306AE4313786AE5C269A?id=195) |
| SIGG | [Società Italiana di Gerontologia e Geriatria](http://www.fism.it/soci/detail.jhtml;jsessionid=59D6723B7947306AE4313786AE5C269A?id=136) |
| SIGO | [Società Italiana di Ginecologia e Ostetricia](http://www.fism.it/soci/detail.jhtml;jsessionid=59D6723B7947306AE4313786AE5C269A?id=137) |
| SIMeL | [Società Italiana di Medicina di Laboratorio](http://www.fism.it/soci/detail.jhtml;jsessionid=59D6723B7947306AE4313786AE5C269A?id=144) |
| SIMG | [Società Italiana di Medicina Generale](http://www.simg.it/) |
| SIMI | [Società Italiana di Medicina Interna](http://www.fism.it/soci/detail.jhtml;jsessionid=59D6723B7947306AE4313786AE5C269A?id=147) |
| SIMeR | [Società Italiana di Medicina Respiratoria](http://www.fism.it/soci/detail.jhtml;jsessionid=59D6723B7947306AE4313786AE5C269A?id=150) |
| SIN | [Società Italiana di Nefrologia](http://www.fism.it/soci/detail.jhtml;jsessionid=59D6723B7947306AE4313786AE5C269A?id=155) |
| SIOeCHCF | [Società Italiana di Otorinolaringologia e Chirurgia Cervico-Facciale](http://www.fism.it/soci/detail.jhtml;jsessionid=59D6723B7947306AE4313786AE5C269A?id=169) |
| SIP | [Società Italiana di Patologia](http://www.unifi.it/istituzioni/sip) |
| SIPAD | [Società Italiana di Patologia Apparato Digerente](http://www.sipad.it/) |
| SIRM | [Società Italiana di Radiologia Medica](http://www.fism.it/soci/detail.jhtml;jsessionid=59D6723B7947306AE4313786AE5C269A?id=180) |
| SIRTS | [Società Italiana di Ricerca e Terapia Sistemica](http://www.spaziopiu.it/sirts/) |
| SIU | [Società Italiana di Urologia](http://www.siu.it/) |
| SIUrO | [Società Italiana di Urologia Oncologica](http://www.fism.it/soci/detail.jhtml;jsessionid=59D6723B7947306AE4313786AE5C269A?id=193) |
| SIGO | [Società Italiana Geriatri Ospedalieri](http://www.sameint.it/sigo/index.html) |
| SIVRQ | [Società Italiana per la Qualità dell'Assistenza Sanitaria](http://www.sivrq.com/) |
| SIT | [Società Italiana Tumori Prevenzione, Diagnosi, Terapia](http://www.societaitalianatumori.it/) |
| SNAMID | [Società Nazionale di Aggiornamento per il Medico di Medicina Generale](http://www.fism.it/soci/detail.jhtml;jsessionid=59D6723B7947306AE4313786AE5C269A?id=211) |
| SIUCP | [Società Unitaria di Colonproctologia](http://www.siucp.org/) |
